# Characterization of Demographic, Clinical, and Laboratory Risk Factors for Stroke in a Tertiary Hospital in Riyadh, Saudi Arabia

**DOI:** 10.7759/cureus.58266

**Published:** 2024-04-14

**Authors:** Adil khalil Hussien, Abdulaziz Khalid Alshehri, Fayez Khalid Alanazi, Abdulaziz mohammed Aljabal, Ahmed Ibrahim Alanazi, Anas Mohammed alqayidi, Ibrahim Hussein Alghamdi

**Affiliations:** 1 College of Medicine, Dar Al Uloom University, Riyadh, SAU; 2 Respiratory Therapy, King Fahad Medical City, Riyadh, SAU; 3 Anesthesia Technology, King Abdulaziz Medical City, Riaydh, SAU; 4 Field Paramedic, Saudi Red Crescent Authority, Riyadh, SAU; 5 Respiratory Therapy, Specialized Medical Center Hospital, Riyadh, SAU; 6 Emergency Medical Services, Prince Sultan Military Medical City, Riyadh, SAU

**Keywords:** ischemic and hemorrhagic stroke, acute hemorrhagic stroke, : ischemic stroke, riyadh population, tertiary medical hospital in riyadh, stroke risk factors, electrolyte abnormality, electrolyte imbalance, electrolyte disturbances, stroke

## Abstract

Background

Stroke is a major cause of death and long-term disability worldwide, with varying incidence and risk factors across different populations. This study aims to analyze demographic, clinical, and laboratory risk factors for stroke among the Saudi Arabian population to enhance the understanding of its behavior and associated mortality.

Methods

In this retrospective cohort study, we analyzed data from 3586 patients diagnosed with hemorrhagic or non-hemorrhagic stroke at King Fahad Medical City from January 1, 2020, to November 11, 2022. We collected data on demographic variables, past medical history, social history, nationality, and laboratory components. Statistical analyses were performed using IBM SPSS Statistics for Windows, Version 27.0. (Armonk, NY: IBM Corp.), with significance set at p<0.05.

Results

The study population was predominantly male (57.86%) and within the age group of 51 to 80 years (58.8%). A significant portion of patients were Saudi nationals (99.6%), with hypertension (50.2%) and diabetes (40.4%) being the most common comorbidities. Laboratory abnormalities related to sodium and potassium levels were strongly linked to mortality rates. Notably, ischemic stroke was the most common type across all age groups, except for patients under age 16, where hemorrhagic stroke was more prevalent.

Conclusions

Our findings reveal significant associations between stroke risk factors and mortality within the Saudi Arabian population, highlighting the impact of hypertension, diabetes, and electrolyte imbalances. The study underscores the need for targeted stroke prevention and management strategies in Saudi Arabia, aligning with global trends to mitigate the burden of this disease.

## Introduction

Stroke ranks as the second most common cause of mortality globally. [1]. Recent years have seen a significant increase in the incidence of stroke and the associated mortality and burdens [2]. In 2020, the United States reported 795,000 stroke cases, with 610,000 being first-time incidents [3]. Stroke remains the primary cause of long-term disability among the elderly population (i.e., those aged 65 and older). Similarly, Europe reported 1.1 million stroke cases and 460,000 deaths in 2017. An epidemiological model predicts a 27% increase in stroke cases for the same region [4].

In comparison, Saudi Arabia reports fewer stroke cases than Western countries, likely due to its generally younger population on average [5]. The incidence of stroke in Saudi Arabia was reported as 29 per 100,000, significantly lower than the 219 per 100,000 reported in the United States by the American Heart Association [6,7]. Nevertheless, a model by Al Senani et al. predicts an increase in first-time stroke incidence among Saudis over the next decade [8]. Our research aims to analyze demographic, clinical, and laboratory risk factors for stroke among Saudis to understand the relationship between laboratory results, clinical history, and mortality.

## Materials and methods

This retrospective cohort study included patients diagnosed with hemorrhagic or non-hemorrhagic stroke (n=3586) at King Fahad Medical City (KFMC) from January 1, 2020, to November 11, 2022. We excluded patients not diagnosed with stroke or those without a complete personal file. We extracted data using the Epic research module and the Slicer Dicer tool (Epic Systems Corporation, Verona, USA), compiling it in a Microsoft Excel 2010 (Microsoft Corporation, Redmond, WA) spreadsheet. We collected variables including demographic data (gender and age), past medical history (diabetes, hypertension, atherosclerotic diseases, and seizure disorders), social history (smoking, marital status), nationality, and laboratory components which were taken after the diagnosis (venous pH, sodium, potassium, albumin, non-high-density lipoprotein (HDL) cholesterol, hemoglobin, platelet count, red blood cell count, sodium bicarbonate in venous blood, and chloride), family history (diabetes, hypertension, stroke, hyperlipidemia, seizure disorder, and no known problems). We also assessed the mortality rate or current patient status (alive, deceased) during the hospital stay, which was defined as the proportion of individuals who died while admitted to the hospital. Patients were divided into four age groups: Group 1 (younger than 16 years), Group 2 (16 to 50 years), Group 3 (51 to 80 years), and Group 4 (older than 80 years).

We received ethical approvals from the Institutional Review Boards of King Fahad Medical City (FWA00018774) and Dar Al Uloom University (PRO23020008), adhering to the Declaration of Helsinki. All personal identifiers were removed for privacy. Data cleaning and editing were performed using Microsoft Excel 2010. We compared our findings with current literature and conducted statistical analyses using IBM SPSS Statistics for Windows, Version 27.0. (Armonk, NY: IBM Corp.). A Chi-square test measured significant differences at a 5% level. We calculated descriptive statistics, inferential statistics, standard deviation, and mean. A p-value of less than 0.05 was considered significant. To control for multiple comparisons, we applied the Bonferroni correction by dividing the significance level by the number of comparisons conducted.

## Results

Table 1 reveals that the study sample comprised 57.86% male patients (n = 2075) and 42.14% female patients (n = 1511), totaling 3586 participants. The majority were in the 51 to 80 years age group, accounting for 58.8% (n = 2108), while the <16 years age group had the lowest representation at 2.3% (n = 83). Participants aged 16 to 50 constituted 27.6% (n = 988), and those over 80 comprised 11.3% (n = 404), with a mean age of 57.8 years across all groups. Saudi nationals represented 99.6% of the subjects (n = 3570), with the remaining 0.4% (n = 16) from other countries. Among the participants, 53.4% were single (n = 1915) and 46.6% were married (n = 1671). Alive subjects constituted 93.9% (n = 3369) of the study, with 6.1% (n = 217) deceased.

**Table 1:** Descriptive statistics

Characteristic		Frequency		Total, n	Percentage
		Male (n)	Female (n)		
Age Group	< 16 Years	47	36	83	2.3%
	16-50 Years	540	448	988	27.6%
	51-80 Years	1278	830	2108	58.8%
	≥80 Years	210	197	407	11.3%
Nationality	Saudi	2071	1499	3570	99.6%
	Others	4	12	16	0.4%
Marital Status	Single	938	977	1915	53.4%
	Married	1137	534	1671	46.6%
Life Status	Alive	1958	1411	3369	93.9%
	Deceased	117	100	217	6.1%

The Chi-square test assessing the relationship between gender and smoking habits found that 95% of participants were non-smokers and 5% were smokers, with male patients significantly more likely to smoke (n = 168) than female patients (n = 10). Approximately half of the participants 50.2% (n = 1799) had a history of hypertension, and less than half 40.4% (n = 1448) had diabetes. A history of previous stroke was reported in 37.1% of patients (n = 1329), atherosclerotic heart disease in 10.4% of patients (n = 373), and seizure disorders in 1.3% of patients (n = 46), with all conditions more prevalent in male patients than female patients (Table 2). Family history showed no significant correlation with stroke, with the highest occurrences in diabetes (1.7% of patients) and hypertension (1.6% of patients), and stroke history reported in only 0.8% of cases. Although these figures were low, they were higher in female patients (Table 3).

**Table 2:** Chi-square tests of gender and disease association Abbreviation: AHD, atherosclerotic heart disease. * Bonferroni difference is statistically significant at p<0.05

Condition		Male Patient (n)	Female Patient (n)	Proportion (%)	P-Value
Smoking	No	1907	1501	95%	0.000
	Yes	168*	10	5%	
History of diabetes	No	1203	935	59.6%	0.019
	Yes	872*	576	40.4%	
History of hypertension	No	1002	785	49.8%	0.030
	Yes	1073*	726	50.2%	
History of AHD	No	1834	1501	89.6%	0.005
	Yes	241*	132	10.4%	
History of seizures	No	2049	1491	98.7%	0.853
	Yes	26	20	1.3%	
History of stroke	No	1303	954	62.9%	0.834
	Yes	772	557	37.1%	

**Table 3:** Chi-square tests of gender and family history association * Bonferroni difference is statistically significant at p<0.05

Family History		Male Patient (n)	Female Patient (n)	Proportion (%)	P-Value
Stroke	No	2064	1493	99.2%	0.070
	Yes	11	16	0.8%	
Diabetes	No	2061	1463	98.3%	0.000
	Yes	14	48*	1.7%	
Hypertension	No	2060	1467	98.4%	0.000
	Yes	15	44*	1.6%	
Seizures	No	2065	1496	99.3%	0.069
	Yes	10	15	0.7%	

**Figure 1:**
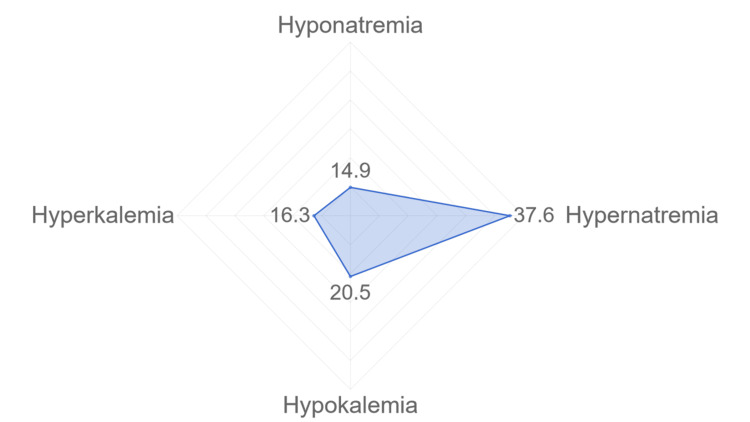
The relationship between the type of electrolyte abnormality and mortality in percentage

The major discrepancies in laboratory results were seen in albumin (1312 male patients, 956 female patients; 63.2% of the total population), sodium (1313 males, 958 females; 63.3% overall), potassium (1306 males, 955 females; 63.1% overall), and hemoglobin levels (1303 males, 956 females, 63% of the total). This was followed by non-HDL cholesterol (793 males, 510 females; 36.3% overall), serum bicarbonate (HCO3; 804 males, 534 females, 37.3% overall), and venous blood gas pH (808 males, 534 females, 37.4% overall). Serum chloride abnormalities were found in 24.7% (n=887) of the subjects. Notably, sodium and potassium level abnormalities were strongly linked to mortality rates during the hospital stay (Figure 1). Among deceased subjects, 14.9% (n=173) experienced hyponatremia, 20.5% (n=155) hypokalemia, 16.3% (n=190) hyperkalemia, and 37.6% (n=136) hypernatremia (Table 4).

**Table 4:** Chi-square tests of gender and laboratory data association Abbreviations: HCO_3_, bicarbonate; HDL, high-density lipoprotein. * Bonferroni difference is statistically significant at p<0.05

Laboratory Test	Status	Male Patient (n)	Female Patient (n)	Proportion (%)	P-Value
Serum Albumin	Normal	763	555	36.8%	0.980
	Abnormal	1312	956	63.2%	
Serum Chloride	Normal	1553	1146	75.3%	0.493
	Abnormal	522	365	24.7%	
Serum HCO3	Normal	1271	977	62.7%	0.037
	Abnormal	804*	534	37.3%	
Serum Hemoglobin	Normal	772	555	37%	0.772
	Abnormal	1303	956	63%	
Serum Sodium	Normal	762	553	36.7%	0.939
	Abnormal	1313	958	63.3%	
Non-HDL-Cholesterol	Normal	1282	1001	63.7%	0.006
	Abnormal	793*	510	36.3%	
Serum Potassium	Normal	769	556	36.9%	0.872
	Abnormal	1306	955	63.1%	
PH Venous Blood gas	Normal	1267	977	62.6%	0.028
	Abnormal	808*	534	37.4%	

**Figure 2:**
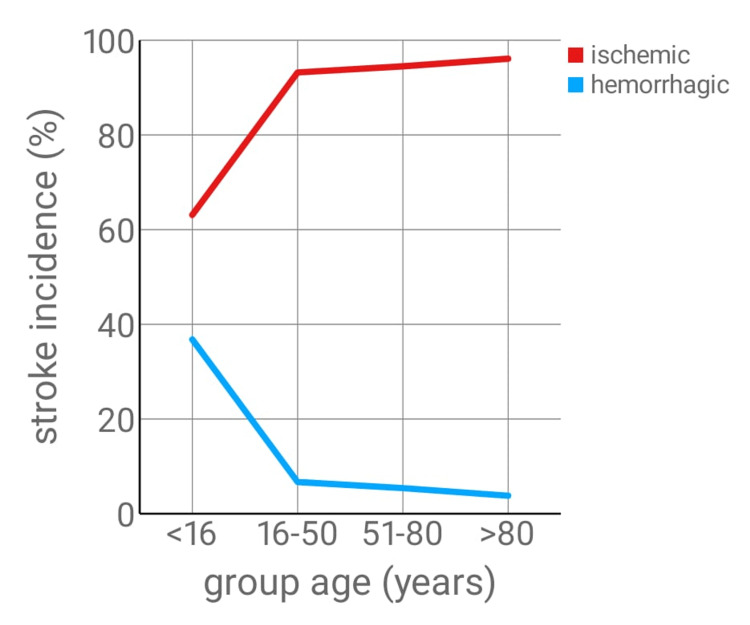
Type of stroke and corresponding age group in percentage (arranged in line chart)

Ischemic stroke was the most common stroke type across all age groups, except in the pediatric population, where 36.8% of patients (n=28) under 16 experienced hemorrhagic strokes. The prevalence of hemorrhagic stroke decreased with age, being replaced by ischemic stroke in older patients (Table 5, Figure 2).

**Table 5:** Type and incidence of stroke by age group

Stroke Type	Age Group in Years			
	<16, n=76	16 – 50, n=939	51 – 80, n=2130	>80, n=443
	n (%)	n (%)	n (%)	n (%)
Hemorrhagic Stroke	28 (36.8%)	63 (6.7%)	117 (5.4%)	17 (3.8%)
Ischemic Stroke	48 (63.1%)	876 (93.2%)	2013 (94.5%)	426 (96.1%)

## Discussion

In our study, we conducted a detailed analysis of laboratory components, medical history, and demographic data related to stroke, aiming to better understand stroke risk factors and behavior within the Saudi Arabian population. Demographically, our findings resonate with current literature, indicating that male patients constitute a larger portion of the stroke population at 57.86% than female patients [9]. The dominance of the Saudi population in our sample at 99.6% could be attributed to the eligibility criteria at KFMC. The mortality rate post-stroke was 6.1%, marginally lower than international figures [10]. Possible explanations include the relatively younger average age of the Saudi population [11], advanced medical facilities reducing mortality rates [12], and the discharge of stabilized patients. Regarding marital status, our data aligns with current trends, showing a slightly higher stroke incidence in unmarried individuals at 53.4% compared to married ones at 46.6% [13].

Our medical history analysis reveals a strong link between stroke and known risk factors such as hypertension, diabetes, and dyslipidemia, aligning with global observations of stroke behavior [14]. Smoking was a risk factor for only 5% of our sample, suggesting potential underreporting, especially in emergencies or when patients could not provide their history [15]. A history of previous strokes was significant in 37.1% of cases, underlining it as a major risk factor [16], whereas only 1.3% reported a history of seizure disorders. Nonetheless, late-life seizures significantly elevate stroke risk [17]. Family history showed no clear patterns.

Regarding laboratory findings, chloride abnormalities were present in 24.7% of patients, mainly within the 51 to 80 age group. This suggests a potential link between chloride imbalance and stroke in the elderly, possibly due to increased rates of acute kidney injury [18,19]. Albumin discrepancies were noted in 63.2% of cases, indicating potential neuroprotection deficits and an elevated inflammatory state [20]. Similarly, sodium and potassium level abnormalities were observed in 63.3% and 63.1% of cases, respectively, and were significantly associated with mortality. Specifically, hypernatremia was present in 37.6% of deceased patients, hyponatremia in 14.9%, hyperkalemia in 16.3%, and hypokalemia in 20.5%. Potassium plays a crucial role in nerve conduction and neurotransmitter release. Hypokalemia can impair these processes, leading to worsened neurological symptoms in stroke patients. This can result in reduced functional recovery and increased disability, which can indirectly contribute to higher mortality rates; similarly, hypernatremia can have detrimental effects on the central nervous system. High sodium levels can disrupt the normal functioning of brain cells by altering osmotic balance and causing cellular shrinkage. This can lead to neurological complications such as seizures, altered mental status, and even cerebral edema. As these complications can increase the risk of mortality in stroke patients, it is safe to say that hypernatremia and hypokalemia propose a lethal combination in stroke patients [21,22]. Thus, we recommend regular electrolyte monitoring, upon admission and after the diagnosis. Moreover, we recommend optimizing fluid therapy in stroke patients and adopting a multidisciplinary approach to stroke care, involving neurologists, nephrologists, intensivists, and other specialists.

HCO_3_ and venous blood gas pH levels were normal in 37.3% and 37.4% of cases, respectively. Although these alterations suggest a metabolic acidotic state impacting cerebrovascular blood flow [23], HCO_3_ levels did not significantly affect mortality, aligning with existing evidence but indicating potential as a prognostic predictor [24]. The serum lipid profile showed a 36.3% rate of non-HDL cholesterol abnormalities, which did not influence mortality rates.

Stroke types varied with age; hemorrhagic strokes accounted for nearly one-third of cases in patients under 16 years (36.8%), shifting progressively from hemorrhagic to ischemic with increasing age, a pattern which is observed within the literature [25,26].

Limitations

This study faces several limitations that warrant consideration. Firstly, its retrospective, single-center design may limit the generalizability of our findings to the broader Saudi Arabian population or other regions. The study's reliance on existing medical records introduces potential biases, including incomplete documentation and social desirability bias, particularly concerning self-reported behaviors like smoking. Additionally, excluding non-Arabic speaking participants or those without a comprehensive medical record may introduce selection bias. Another significant limitation is the lack of detailed analysis of the impact of lifestyle factors, socioeconomic status, and access to healthcare services, which are known to influence stroke risk and outcomes. Furthermore, the study did not account for genetic factors that could be crucial in stroke susceptibility and recovery. The cross-sectional nature of the data collection limits our ability to establish causality between the identified risk factors and stroke occurrence. Lastly, potential advancements in stroke management and healthcare access during the study period were not analyzed, which could affect the interpretation of the results concerning temporal trends in stroke care and outcomes.

## Conclusions

Our research provides a robust overview of stroke risk factors and behaviors among the Saudi Arabian population. We found that patients with hypokalemia or hypernatremia faced higher mortality risks. Additionally, hypertension, diabetes, and a history of previous strokes emerged as significant risk factors. These findings align with global trends and enrich the knowledge base for stroke prevention, management, and future research in Saudi Arabia. This research highlights the necessity of implementing specific strategies for stroke prevention and management in Saudi Arabia, in line with worldwide efforts to reduce the impact of this condition.

## References

[1] Sarvari S, Moakedi F, Hone E, Simpkins JW, Ren X (2020). Mechanisms in blood-brain barrier opening and metabolism-challenged cerebrovascular ischemia with emphasis on ischemic stroke. Metab Brain Dis.

[2] GBD 2019 Stroke Collaborators (2021). Global, regional, and national burden of stroke and its risk factors, 1990-2019: a systematic analysis for the Global Burden of Disease Study 2019. Lancet Neurol.

[3] (2024). Stroke Facts. https://www.cdc.gov/stroke/facts.htm.

[4] Wafa HA, Wolfe CD, Emmett E, Roth GA, Johnson CO, Wang Y (2020). Burden of stroke in Europe: thirty-year projections of incidence, prevalence, deaths, and disability-adjusted life years. Stroke.

[5] al Rajeh S, Awada A, Niazi G, Larbi E (1993). Stroke in a Saudi Arabian National Guard community. Analysis of 500 consecutive cases from a population-based hospital. Stroke.

[6] Alqahtani BA, Alenazi AM, Hoover JC, Alshehri MM, Alghamdi MS, Osailan AM, Khunti K (2020). Incidence of stroke among Saudi population: a systematic review and meta-analysis. Neurol Sci.

[7] Tsao CW, Aday AW, Almarzooq ZI (2022). Heart disease and stroke statistics-2022 update: A report from the American Heart Association. Circulation.

[8] Al-Senani F, Al-Johani M, Salawati M (2020). An epidemiological model for first stroke in Saudi Arabia. J Stroke Cerebrovasc Dis.

[9] Abdu H, Seyoum G (2022). Sex differences in stroke risk factors, clinical profiles, and in-hospital outcomes among stroke patients admitted to the medical ward of Dessie Comprehensive Specialized Hospital, Northeast Ethiopia. Degener Neurol Neuromuscul Dis.

[10] Varona JF, Bermejo F, Guerra JM, Molina JA (2004). Long-term prognosis of ischemic stroke in young adults. Study of 272 cases. J Neurol.

[11] Basri R, Issrani R, Hua Gan S, Prabhu N, Khursheed Alam M (2021). Burden of stroke in the Kingdom of Saudi Arabia: a soaring epidemic. Saudi Pharm J.

[12] Lackland DT, Roccella EJ, Deutsch AF (2014). Factors influencing the decline in stroke mortality: a statement from the American Heart Association/American Stroke Association. Stroke.

[13] Liu Q, Wang X, Wang Y (2018). Association between marriage and outcomes in patients with acute ischemic stroke. J Neurol.

[14] McFarlane SI, Sica DA, Sowers JR (2005). Stroke in patients with diabetes and hypertension. J Clin Hypertens (Greenwich).

[15] Si Larbi MT, Al Mangour W, Saba I, Al Naqeb D, Faisal ZS, Omar S, Ibrahim F (2021). Ischemic and non-ischemic stroke in young adults - a look at risk factors and outcome in a developing country. Cureus.

[16] Chohan SA, Venkatesh PK, How CH (2019). Long-term complications of stroke and secondary prevention: an overview for primary care physicians. Singapore Med J.

[17] Ben-Menachem E (2005). Epilepsy as a warning sign for stroke. Epilepsy Curr.

[18] Qureshi AI, Ma X, Huang W (2022). Early hyperchloremia and outcomes after severe traumatic brain injury: analysis of resuscitation outcomes consortium hypertonic saline trial. Crit Care Explor.

[19] Yokota LG, Sampaio BM, Rocha EP, Balbi AL, Sousa Prado IR, Ponce D (2018). Acute kidney injury in elderly patients: narrative review on incidence, risk factors, and mortality. Int J Nephrol Renovasc Dis.

[20] Dziedzic T, Pera J, Slowik A, Gryz-Kurek EA, Szczudlik A (2007). Hypoalbuminemia in acute ischemic stroke patients: frequency and correlates. Eur J Clin Nutr.

[21] Gao F, Wang CT, Chen C, Guo X, Yang LH, Ma XC, Han JF (2017). Effect of hypokalemia on functional outcome at 3 months post-stroke among first-ever acute ischemic stroke patients. Med Sci Monit.

[22] Huang WY, Weng WC, Peng TI (2012). Association of hyponatremia in acute stroke stage with three-year mortality in patients with first-ever ischemic stroke. Cerebrovasc Dis.

[23] Caldwell HG, Carr JM, Minhas JS, Swenson ER, Ainslie PN (2021). Acid-base balance and cerebrovascular regulation. J Physiol.

[24] Huang X, Zhang Y (2023). Relationship between serum bicarbonate levels and the risk of death within 30 days in ICU patients with acute ischemic stroke. Front Neurol.

[25] Namaganda P, Nakibuuka J, Kaddumukasa M, Katabira E (2022). Stroke in young adults, stroke types and risk factors: a case control study. BMC Neurol.

[26] Fullerton HJ, Wu YW, Zhao S, Johnston SC (2003). Risk of stroke in children: ethnic and gender disparities. Neurology.

